# Structural studies suggest aggregation as one of the modes of action for teixobactin†
†Electronic supplementary information (ESI) available: Additional figures. NMR titration data. FRET assays. NMR spectra. NMR relaxation data. PDB of the structure of teixobactin in DPC micelles. See DOI: 10.1039/c8sc03655a


**DOI:** 10.1039/c8sc03655a

**Published:** 2018-09-20

**Authors:** Carl Öster, Grzegorz P. Walkowiak, Dallas E. Hughes, Amy L. Spoering, Aaron J. Peoples, Anita C. Catherwood, Julie A. Tod, Adrian J. Lloyd, Torsten Herrmann, Kim Lewis, Christopher G. Dowson, Józef R. Lewandowski

**Affiliations:** a Department of Chemistry , University of Warwick , Coventry , CV4 7AL , UK . Email: j.r.lewandowski@warwick.ac.uk; b School of Life Sciences , University of Warwick , Coventry , CV4 7AL , UK; c NovoBiotic Pharmaceuticals , Cambridge , MA 02138 , USA; d Univ. Grenoble Alpes , CNRS , CEA , IBS , F-38000 Grenoble , France; e Antimicrobial Discovery Center , Northeastern University , Department of Biology , Boston , MA 02115 , USA

## Abstract

Teixobactin is a new promising antibiotic that targets cell wall biosynthesis by binding to lipid II and has no detectable resistance thanks to its unique but yet not fully understood mechanism of operation. To aid in the structure-based design of teixobactin analogues with improved pharmacological properties, we present a 3D structure of native teixobactin in membrane mimetics and characterise its binding to lipid II through a combination of solution NMR and fast (90 kHz) magic angle spinning solid state NMR. In NMR titrations, we observe a pattern strongly suggesting interactions between the backbone of the C-terminal “cage” and the pyrophosphate moiety in lipid II. We find that the N-terminal part of teixobactin does not only act as a membrane anchor, as previously thought, but is actively involved in binding. Moreover, teixobactin forms a well-structured and specific complex with lipid II, where the N-terminal part of teixobactin assumes a β conformation that is highly prone to aggregation, which likely contributes to the antibiotic's high bactericidal efficiency. Overall, our study provides several new clues to teixobactin's modes of action.

## Abstract 2

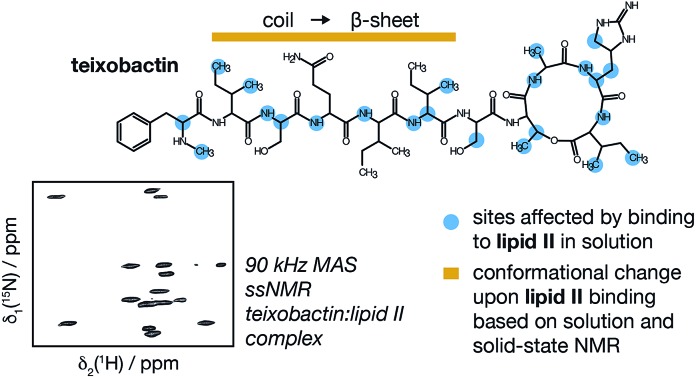
Combination of solution and solid state NMR yields a molecular level view of the interactions between antibiotic teixobactin and bacterial cell wall building block lipid II.

## Introduction

**Fig. 1:**
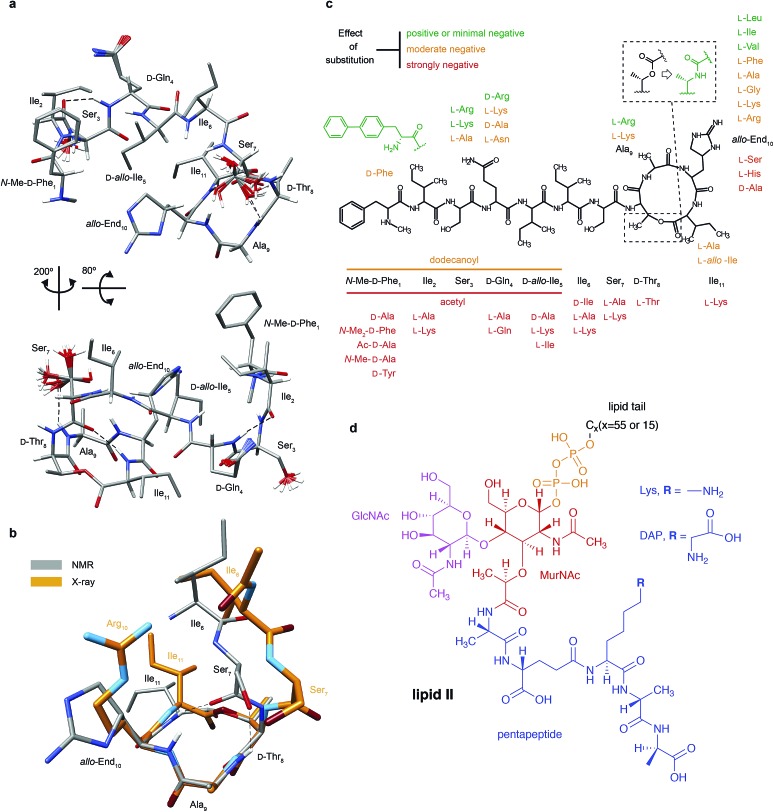
Structural details of teixobactin. (a) Solution NMR structure of teixobactin in DPC micelles presented from two different views. The 20 lowest energy structures are shown. For clarity all of the protons except H^N^, H^α^ and hydroxyl protons were omitted from the figure (a PDB file of the structure is provided in the ESI†). (b) Comparison of the “pyrophosphate binding cage” in the solution NMR structure of native teixobactin with the crystal structure of the teixobactin analogue Ac-Δ_1–5_Arg_10_-teixobactin.^14^ The grey backbone represents a single conformer from the solution NMR structure and orange represents the crystal structure. Note that the intramolecular hydrogen bond between Ser_7_ and Ala_9_ is present only in a subset of the 20 lowest energy structures in the NMR structure. (c) A summary of the effect of various substitutions on the activity of teixobactin^10^,^12^–^18^ shown with the chemical structure of teixobactin as reference. The substitutions with a positive or very small effect on the antibacterial activity are indicated in green font. The substitutions that have a moderate effect are indicated in orange font. The substitutions that have a strong negative effect are indicated in red font. (d) Chemical structure of lipid II with building blocks highlighted in different colours: GlcNAc indicates *N*-acetylglucosamine (magenta), MurNAc indicates *N*-acetylmuramic acid (red), pentapeptide signifies l-Ala-d-Glu-l-Lys/DAP-d-Ala-d-Ala (blue), *C*_*X*_ represents the lipid tail with *X* indicating the number of carbons in the chain. There were two variants of lipid II used in this study: Gram-positive (lysine in position 3 in the pentapeptide) and Gram-negative (diaminopimelic acid (DAP) in position 3 in the pentapeptide), distinguished by the different substituent R. The Gram-negative variant of lipid II used in this study had a native C_55_ lipid chain. The Gram-positive lipid II variant was rendered water soluble by shortening the lipid tail to C_15_.

The majority of antibiotics currently in clinical use have been discovered by screening cultivable soil bacteria. However, around 99% of microorganisms are uncultured, meaning that they do not grow under laboratory conditions. Recently, several methods to access potential antimicrobial compounds from uncultured microorganisms were developed,^1^–^4^ of which the iChip technology led to the discovery of teixobactin from a new species of β-proteobacteria, *Eleftheria terrae*.^5^ Teixobactin was shown to have very good activity against many difficult-to-treat bacterial pathogens, including *Mycobacterium tuberculosis*, and no resistant mutants were obtained *in vitro* with various bacteria. Teixobactin binds to both lipid II and lipid III and thus simultaneously inhibits the biosynthesis of peptidoglycan and teichoic acids, triggering synergistic effects leading to increased cell wall damage and delocalization of autolysins.^5^,^6^ Additionally, teixobactin, in contrast to vancomycin, does not bind mature peptidoglycan, which enables the efficient killing of bacteria with increased cell wall density such as vancomycin-intermediate *Staphylococcus aureus* (VISA) against which vancomycin is ineffective.^6^ The biosyntheses of both peptidoglycan and teichoic acids rely on a common lipid carrier, bactoprenol, C_55_-P, which is linked in both cases to a disaccharide unit *via* a pyrophosphate bridge. In this work we focus on the peptidoglycan precursor lipid II, which, apart from the lipid carrier and the pyrophosphate, contains the main building blocks of peptidoglycan; *N*-acetylmuramic acid, *N*-acetylglucosamine and a pentapeptide (l-Ala-d-Glu-l-Lys/DAP-d-Ala-d-Ala; see Fig. 1d). Lipid II biosynthesis varies, typically within the pentapeptide at position three between Gram-positive bacteria possessing lysine and Gram-negative bacteria diaminopimelic acid (DAP). *Mycobacteria* spp. possess DAP and can additionally modify *N*-acetyl- to *N*-glycolyl-muramic acid.^7^ Several other modifications to the peptide stem or glycan strands have been discovered and are discussed elsewhere.^8^,^9^ Teixobactin is expected to interact with the pyrophosphate of lipid II and it can thus bind both Gram-positive and Gram-negative variants of lipid II, regardless of any modifications to the sugars or peptides.^5^ Since the discovery of teixobactin, its biosynthetic pathway and the identification of lipid II as a primary target, a large number of studies have been conducted to gain more understanding on the mode of action of teixobactin, with the goal of developing analogues with better pharmacological properties. Several groups have synthesized teixobactin analogues to investigate the roles of the different residues in teixobactin and potentially find active compounds that are easier to make and better suited for clinical use (see Fig. 1c for a summary of the effect on antimicrobial activity by substitution of amino acids). An NMR study of seven analogues showed the importance of the d-amino acids for activity.^10^ The residue in position 10, l-allo-enduracididine, is a non-proteinogenic amino acid, which has been difficult to synthesise and hence replacing it with a naturally available amino acid is an attractive approach. Arg_10_teixobactin^11^,^12^ and Lys_10_teixobactin^12^ showed good activity and recently several other teixobactin analogues with different alternatives in position 10 were synthesised and found to have good activity against methicillin-resistant *Staphylococcus aureus* (MRSA), *Staphylococcus epidermidis* and *Bacillus subtilis*.^13^ Structural details of teixobactin analogues in isolation and some initial studies involving teixobactin analogues and mimics of lipid II in dimethyl sulfoxide (DMSO) and molecular dynamics simulations exploring the binding space were reported in the literature.^10^,^13^–^15^,^19^ However, to date no structure of wild type teixobactin and no high resolution structural information on the interactions with actual lipid II have been reported. Here we use a combination of solution and fast magic angle spinning (MAS) solid-state NMR complemented by a fluorescence based assay to obtain insights on the interactions of wild type teixobactin with both Gram-negative and Gram-positive lipid II and conformational changes of teixobactin induced by binding to lipid II. We find that the majority of the sites interacting with lipid II are located on the backbone of teixobactin and that teixobactin assumes an aggregation prone β-strand conformation for residues 2–6 upon binding lipid II. To mimic the natural membrane environment of lipid II we used dodecylphosphocholine (DPC) micelles but we also consider interactions of teixobactin with a water soluble lipid II in aqueous solution. Our data provide a rationale for understanding the behaviour of numerous analogues of teixobactin and valuable insights that should further guide efforts to engineer teixobactin analogues.

## Results

### Teixobactin in solution

Since teixobactin binds lipid II in the cytoplasmic membrane of bacteria, we investigated its conformation in a simulated membrane using DPC micelles. The 20 lowest energy structures of teixobactin in DPC micelles in phosphate buffer determined by solution NMR are shown in Fig. 1a (see ESI Table 1† for details). The structures are overlaid with a crystal structure of the teixobactin analogue Ac-Δ_1–5_Arg_10_-teixobactin as a hydrochloride salt^14^ (Fig. 1b). The proposed pyrophosphate binding cage formed by residues 8–11 is nearly identical in the NMR and crystal structures. The most obvious differences between the structures are that the bond between Ser_7_C′ and d-Thr_8_N points in different directions and the side-chain of residue 10, where in the analogue the allo-enduracididine has been replaced by arginine. Additionally, the N-termini are different in the structures since residues 1–5 were replaced by an acetyl group in the crystal structure construct.^14^

Structures of several analogues including teixobactin-Arg_10_ were also solved in DMSO by solution NMR.^10^ In the structure of teixobactin-Arg_10_ and in contrast to the structure of native teixobactin in DPC micelles, presented here, residues 1–7 are largely unstructured. This discrepancy is likely due to DMSO being used as a solvent for the teixobactin analogues, as DMSO is known to destabilise the secondary structure and promote disorder.^20^ Since, similar to other lipid II binding antibiotics,^21^ the solvent environment appears to have a potentially large effect on the structure of teixobactin, we have also performed solution NMR experiments on teixobactin in a phosphate buffer in the absence of micelles. A comparison of the chemical shifts in the two media (*i.e.* aqueous solution in the presence and absence of DPC micelles) suggests that no large conformation change (except a slight change near residues 1 & 6) occurs between them with most changes being attributable to hydrophobic interactions between teixobactin and DPC micelles (with residues 2, 6, 7 and 11 being most likely to be involved in or influenced by binding to the micelle; see Fig. ESI1†).

It has been previously suggested that teixobactin in aqueous solution binds lipid II in a 2 : 1 ratio,^5^ which could be potentially explained if teixobactin dimerizes under these conditions. To investigate the oligomeric state of teixobactin we measured ^15^N NMR relaxation in aqueous solution (see Fig. ESI3, Table ESI2†). The correlation time (*τ*_c_) for the overall tumbling and subsequently the approximate size of the molecule were estimated from ^15^N relaxation times. The relaxation measurements indicate that teixobactin is not monomeric in aqueous solution. The correlation time calculated from the relaxation times corresponds much better to a dimer or a trimer than to a monomer (see Fig. ESI3, Table ESI3†). The relaxation rates are also consistent with considerable local flexibility of the N-terminal residues (order parameter, *S*^2^, of 0.4–0.6). Since the N-terminus appears quite mobile we have also considered whether the increased apparent hydrodynamic radius of teixobactin in solution could be potentially explained by a monomeric teixobactin where the molecule assumes a more extended conformation as observed for some teixobactin analogues in DMSO (though minimal changes in chemical shifts between teixobactin in DPC micelles and in aqueous solution do not support such interpretation). However, all of the extended monomers we generated resulted in underestimated values for *τ*_c_ (see Fig. ESI3, Table ESI3†). Because DPC micelles dominate the tumbling a similar method cannot be used to estimate the oligomeric state of teixobactin in DPC micelles (see Fig. ESI4 and Table ESI2† for relaxation data). Due to the lack of any plausible intermolecular NOEs, teixobactin in DPC micelles is assumed to be monomeric for structure calculations, which is a general assumption made for peptides in DPC micelles.

### Teixobactin–lipid II interactions

Whereas teixobactin did not exhibit any particular propensity for aggregation in DPC micelles, we found that addition of lipid II resulted in rapid aggregation with peaks disappearing in the solution NMR spectra due to the large size of the aggregates. We observed the same behaviour when a water soluble variant of lipid II, C_15_ lipid II as opposed to the native C_55_ lipid II, was added to teixobactin in aqueous solution in the absence of DPC micelles. This suggests that the aggregation is induced by interactions between teixobactin and lipid II and does not require a membrane environment. Previously, adding various amounts of DMSO was demonstrated to facilitate obtaining soluble antibiotic–lipid II complexes and to prevent their aggregation and precipitation.^22^,^23^ However, we observe strong aggregation in the teixobactin–lipid II complex even in solutions containing 80% DMSO. Since in our case DMSO does not alleviate the aggregation and because of its high potential for artificially introducing disorder and suppressing potentially important interactions, we have avoided it in further experiments.

To gain insights into the aggregation process we performed a Fluorescence Resonance Energy Transfer (FRET) assay on a series of lipid II binders. Among several tested antibiotics including teixobactin, ramoplanin, deoxyactagardin B, mersacidin and vancomycin, only ramoplanin and teixobactin promoted aggregation of lipid II molecules (see Fig. ESI5†).

**Fig. 2:**
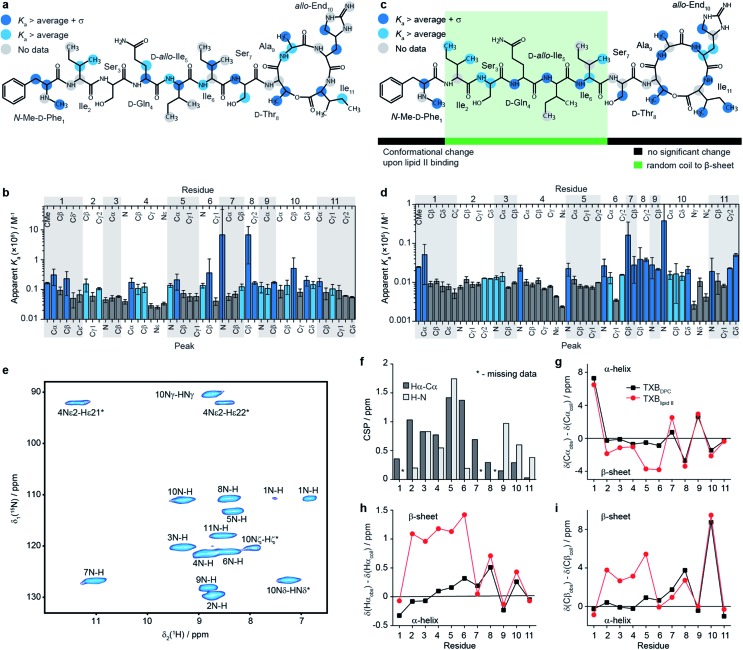
Characterisation of the teixobactin–lipid II complex. (a–d) Results of solution NMR titrations of teixobactin with a water soluble C_15_ variant of Gram-positive lipid II in aqueous solution (a, b) and with native C_55_ Gram-negative lipid II in DPC micelles (c, d). Panels b and d show the measured apparent *K*_a_ values and plotted onto the chemical structure of teixobactin in a and c. Dark blue indicates sites with apparent *K*_a_ values higher than the average plus one standard deviation and light blue indicates sites with apparent *K*_a_ values higher than the average. Dark grey indicates sites with a below average apparent *K*_a_ (b and d). In a and c grey circles indicate sites for which data are not available. (e) 2D ^1^H-^15^N solid state NMR correlation spectrum of sedimented [U^13^C-^15^N]teixobactin in complex with natural abundance Gram-negative lipid II in DPC micelles acquired at 600 MHz ^1^H Larmor frequency and 90 kHz magic angle spinning frequency. In panel e star indicates peaks folded in the ^15^N dimension. The two resonances for residue 1 are due to the zwitterionic form of *N*-Me-d-Phe_1_ being the major form at pH 6.5. (f) ^1^H-^15^N (light grey) and ^1^H^α-13^C^α^ (dark grey) chemical shift perturbations between TXB_DPC_ and TXB_lipid II_. Comparisons of secondary ^13^C^α^ (g), ^1^H^α^ (h) and ^13^C^β^ (i) chemical shifts between TXB_DPC_ (black squares) and TXB_lipid II_ (red circles).

In order to determine whether the aggregation occurs due to nonspecific or specific interactions we have also sedimented the soluble aggregates formed between teixobactin and lipid II in DPC micelles using ultracentrifugation and performed fast magic angle spinning (MAS) solid-state NMR experiments. We observed only one set of resonances in the solid state NMR spectra, consistent with the presence of a single dominant conformer in the sedimented complex (see Fig. 2e and ESI6, 7†). The narrow, under the applied conditions, linewidths (at 90 kHz spinning) ^1^H 0.2–0.4 ppm (120–240 Hz), ^13^C 0.4–0.6 ppm (60–90 Hz) and ^15^N ∼0.7 ppm (45 Hz) indicate unambiguously that the aggregates are homogeneous in nature and are driven by specific rather than nonspecific interactions.^24^ Note that even moderate local disorder would be reflected in substantial broadening.

Interestingly, in a study by Parmar *et al.*^15^ no aggregation was observed for teixobactin analogues in DMSO in the presence of geranyl pyrophosphate, a lipid II mimic possessing a pyrophosphate and C_10_ isoprenyl chain but no sugar moieties or the peptide stem. This result combined with our observations suggests that the interaction with the sugar moieties of lipid II may play a role in the aggregation process for the teixobactin : lipid II complex.

To obtain a site-specific view of the effect of the binding of teixobactin to lipid II we have performed NMR titrations in solution, where changes in teixobactin spectra were followed as a function of lipid II concentration. Upon addition of lipid II we did not observe changes in chemical shifts for any peaks but rather a decrease in intensities of the peaks, which is behaviour characteristic of slow exchange with dissociation constants, *K*_d_, in the low μM to nM range. To determine binding affinities in the presence of slow exchange, typically, one follows the build-up of peak intensities of the bound form.^25^ This was not possible in the case of lipid II titrations because the resonances for the bound form of teixobactin were broadened beyond detection due to aggregation. Instead, we fitted intensities of the peaks from free teixobactin as a function of lipid II concentration to extract apparent association constants^26^ (*K*_a_ is the inverse of *K*_d_; see eqn (1) in methods, see Fig. ESI8–11, Tables ESI4, 5†). It should be noted that the *K*_a_ values obtained by these fits may be higher than the actual *K*_a_ values due to the effect aggregation has on the NMR spectra. However, even if the calculated *K*_a_ values may not be quantitative, they yield qualitative information on which sites of teixobactin are the most affected by the binding of lipid II either due to interaction or conformational change upon interaction. We performed the experiments using both native Gram-negative lipid II (diaminopimelic acid in position 3 of the peptide stem, see Fig. 1d) with the full C_55_ lipid tail in DPC micelles (in the following we refer to this sample as TXB_DPC_; see Fig. 2e) and a water soluble version of Gram-positive lipid II (lysine in position 3 of the peptide stem, see Fig. 1d) with a C_15_ lipid tail in aqueous solution (in the following we refer to this sample as TXB_aqueous_; see Fig. 2c). To facilitate the analysis, in Fig. 2b–e we have highlighted in light blue all the sites for which the apparent *K*_a_ is larger than the average apparent *K*_a_ and in dark blue all the sites for which apparent *K*_a_ is larger than the average apparent *K*_a_ plus one standard deviation.

The apparent *K*_a_ values for TXB_aqueous_ are approximately one order of magnitude larger than in TXB_DPC_, which might indicate that the peptide stem of lipid II plays a role in the binding or that the addition of DPC has an effect on the binding strength. However, due to the above-mentioned ambiguity of the *K*_a_ values obtained from fitting of disappearing peaks we limit ourselves to qualitative comparisons only.

Although we see an overall difference between the binding strength in TXB_DPC_ and in TXB_aqueous_, the binding mode seems to be very similar in both cases. The strongest effect appears in the same parts of teixobactin in both media. The majority of the sites strongly affected by the titration of lipid II are backbone atoms. In fact, there are backbone atoms showing strong affinity in all residues except 2 and 3; for residue 2 data are missing for N-H (TXB_aqueous_) and C^α^-H^α^ (TXB_aqueous_ and TXB_DPC_), for residue 3 N-H and C^α^-H^α^ show apparent *K*_a_ above average (light blue in Fig. 2a–d) but not significantly above average (dark blue in Fig. 2b–e) for TXB_DPC_. For the side chains there are no strongly affected sites in residues 2–5, the most strongly affected side-chains include: *N*-Me-d-Phe_1_C^Me^, *N*-Me-d-Phe_1_C^β^ (TXB_aqueous_ only), l-Ile_6_C^β^, l-Ser_7_C^β^ (TXB_DPC_ only, above average *K*_a_ for TXB_aqueous_), d-Thr_8_C^β^, d-Thr_8_C^γ2^, l-End_10_C^β^, l-End_10_C^δ^ (TXB_aqueous_ only, above average apparent *K*_a_ for TXB_DPC_), l-Ile_11_C^γ2^(TXB_DPC_ only), and l-Ile_11_C^δ^ (TXB_DPC_ only). Most sites that show strong apparent affinity (apparent *K*_a_ above average plus standard deviation) in either TXB_aqueous_ or TXB_DPC_ show at least apparent affinity above average in the other. The only exceptions are the *N*-Me-d-Phe_1_C^β^ (strong apparent affinity in TXB_aqueous_ only) and the side chains of residue 11, where C^γ2^ and C^δ^ show strong affinity in TXB_DPC_ but not in TXB_aqueous_. In the backbone of l-Ile_4_ C^α^ shows strong affinity in TXB_aqueous_ but not in TXB_DPC_ and the NH shows strong affinity in TXB_DPC_ but not in TXB_aqueous_. The deviations for the C^β^ of *N*-Me-d-Phe_1_ and for the side-chains of l-Ile_11_ can possibly be explained by hydrophobic interactions with the DPC micelles which are affected due to conformational changes occurring in teixobactin when lipid II is added. The phenyl group of *N*-Me-d-Phe_1_ has previously been suggested to interact through hydrophobic interactions with the membrane and the lipid tail of lipid II.^27^ The deviations for C^α^ and NH for residue 4 could possibly indicate slightly different backbone conformations in the two samples: NH of residue 4 is among those peaks showing largest chemical shift changes between TXB_aqueous_ and TXB_DPC_ (see Fig. ESI1†). It is also possible that the different peptide stems between the Gram-negative lipid II used in the titrations to TXB_DPC_ and the Gram-positive lipid II used in the titrations to TXB_aqueous_ have some effect on which sites are mostly affected but the available data are inconclusive in this respect.

In our titrations we see a strong effect on the backbone for residues 4–6, which is not seen in the titration of the teixobactin-Ala_10_ analogue with geranyl pyrophosphate^15^ (lacking the sugars and peptide chain compared to the native lipid II). Absence of indication of any specific interaction with geranyl pyrophosphate for these residues suggests that they might be involved in interactions with sugar moieties and possibly parts of the peptide stem in native lipid II.

As mentioned previously due to aggregation we could not detect the bound form of teixobactin by solution NMR but we could study it by fast MAS solid state NMR, which is not limited by the large size of the aggregates.^24^,^28^–^30^ Fig. 2e shows a cross-polarization (CP) based ^1^H-^15^N correlation spectrum of [U-^13^C,^15^N]teixobactin in a sedimented complex with unlabelled Gram-negative lipid II in the presence of DPC (in the following we refer to this sample as TXB_lipid II_). The high quality spectra obtained by solid state NMR allowed for complete backbone assignments of TXB_lipid II_ and almost complete side-chain assignments (the aromatic group of residue 1 was not completely assigned).

To gain some insights into the intermolecular interactions and conformation of teixobactin in the complex we calculated chemical shift perturbations (CSPs, eqn (4) in methods) between TXB_DPC_ (solution) and TXB_lipid II_ (solid; see Fig. 2f) and secondary chemical shifts (Fig. 2g–i). The ^1^H-^15^N CSPs are sensitive to both binding and conformational changes and ^1^H^α-13^C^α^ CSPs are mostly sensitive to conformational changes. The large ^1^H-^15^N CSPs for residues 3–5 and 9–10 confirm the view available from the titrations in solution that the binding of teixobactin to lipid II extends beyond the pyrophosphate binding cage formed by residues 8–11. The ^1^H^α-13^C^α^ CSPs suggest that notable conformational changes take place for residues 2–7 with the conformation of residues 8–11 being very similar to what we observe in solution for isolated teixobactin. The conformational changes are more evident from the secondary chemical shifts, which report on secondary structure deviations from random coil. Combined analysis of ^13^C^α^, ^1^H^α^ and ^13^C^β^ secondary chemical shifts suggests that residues 2–6 rearrange from a random coil like conformation to a more extended β-strand like conformation upon binding to lipid II. Again, the back-bone conformation for residues 8–11 appears largely unchanged upon complex formation.

## Discussion

The charged side chain of the l-allo-enduracididine amino acid at position 10 was initially thought to be important for the antimicrobial activity and hence most of the initial work focused on analogues of teixobactin with positively charged side chains: End_10_Arg and End_10_Lys variants both displayed antimicrobial activity but not as good as the wild type teixobactin.^12^,^13^ In the X-ray structure of Ac-Δ_1–5_Arg_10_-teixobactin analogue the guanidinium side chain of Arg_10_ formed a hydrogen bond to the chloride ion^14^ (mimicking the pyrophosphate of lipid II). Interestingly, according to our titrations there is no strong interaction between the side chain nitrogens of residue 10 and lipid II. However if we look at chemical shift changes between TXB_DPC_ and TXB_lipid II_ the CSPs are large for the side-chain NH groups; 0.44, 1.36 and 0.74 ppm for N^γ^, N^δ^ and N^ζ^ respectively, which, compared to the CSPs for the backbone amides, would suggest that N^δ^ is one of the most affected nitrogen sites. The side-chains of d-Gln_4_ show even larger chemical shift changes (the chemical shift in TXB_DPC_ is 112 ppm for the N^ε2^ and 41.1 ppm in TXB_lipid II_ and the ^1^H chemical shifts for H^ε21^ and H^ε22^ are 6.8 and 7.5 ppm in TXB_DPC_ and 8.5 and 11.4 ppm in TXB_lipid II_). The large chemical shift changes for side-chain nitrogen can be explained by conformational changes due to interactions with the carbon side-chains in the case of End_10_. The unusual chemical shift for d-Gln_4_ side-chain nitrogen suggests interactions with charged moieties, *e.g.*d-Glu in the pentapeptide of lipid II: the only sites with similar chemical shifts for the systems deposited in BMRB occur for glutamines in close contact with charged, mostly negatively charged, side chains. The fact that a charged side chain for residue 10 is not required was recently demonstrated by creating teixobactin analogues where l-allo-End_10_ was replaced by residues with hydrophobic side chains that exhibited antimicrobial activity^15^,^16^ even at the level matching the activity of wild type teixobactin.^15^ Interestingly, the best performing analogues are the ones where the side chain can assume a similar conformation to the one found for interacting sites in our titrations, End_10_Ile and End_10_Leu,^15^ suggesting that for the side chain of residue 10 the shape is more important than the charge or hydrophobicity. The other residues of teixobactin whose replacement in analogues did not lead to an overly detrimental effect on the antimicrobial activity include Ser_3_ and Ala_9_. Residue 3 did not show any significant interaction according to our titrations but backbone conformational changes of residue 3 appear important for the activity. According to our titrations the side-chain of residue 9 is strongly involved in the binding to lipid II, however it has been shown that the alanine in position 9 can be exchanged to a lysine or arginine without significant loss of antimicrobial effect^14^ (see Fig. 1c). This would suggest that the backbone of residue 9 is involved in the interactions but it is not important that the side-chain is hydrophobic. The N-terminus of teixobactin has been suggested to function primarily as an anchoring point to the cell membrane, however our data suggest that it could have a more involved role in the bactericidal mechanism of teixobactin by promoting aggregation, which may explain the lower activity seen in analogues where residues 1–5 were replaced by a dodecanoyl group^14^ and why substitution of residues 1, 2, 5 and 6 is not well tolerated.^16^ Our data suggest that most of the sites except the aromatic ring in the first residue are involved in the interactions with lipid II both in the presence and absence of DPC micelles. The removal of the methyl group interacting with lipid II leads only to a modest reduction of the antibacterial activity^17^ but loss of the positive charge or replacement of the methyl group with a bulkier hydrophobic group has much greater negative impact.^31^ In the context of our study this could suggest that bulky substituents interfere with the interaction with lipid II. The removal of the aromatic ring, that is not interacting with lipid II according to our data, leads to loss of antibacterial activity altogether but substitution with larger hydrophobic groups has a positive effect.^16^,^27^ That larger hydrophobic groups do not decrease the activity is consistent with the side chain of residue 1 not being involved in binding to lipid II but rather contributing to anchoring to the membrane. On the other hand, the aromatic ring could also aid the aggregation process. The general chemical shift changes indicative of β-strand formation for residues 2–6 and the assembly of large soluble aggregates upon interaction of teixobactin and lipid II suggest that these residues may aid in the aggregation process (exposed β-strands are involved in an important mode for protein–protein interactions linked to aggregation^32^), perhaps by fibril formation, as was previously reported for the ramoplanin : lipid II complex^22^ or the type of aggregation observed for the nisin : lipid II complex^33^,^34^ (though the precise nature of this specific aggregate is unclear and requires further studies). This highlights that flexibility of the N-terminal part of teixobactin is important for its biological activity, as has been suggested based on studies of several analogues of teixobactin involving substitutions of amino acids in this part.^10^ However, our data suggest that the conclusion of the mentioned study should be slightly modified: it is not the disorder that is the key (the N-terminus is mobile but well-ordered in our structure of teixobactin in DPC micelles) but rather the range of accessible conformations promoting intermolecular rather than intramolecular β-sheet formation.

Aggregation makes it difficult to study structural aspects of the teixobactin : lipid II complex using conventional methods such as X-ray crystallography and solution NMR. However, we have shown that ultra-fast MAS solid state NMR can be used to gain valuable site-specific information in this system. More investigations into the teixobactin : lipid II complex using isotope labelled lipid II are required to resolve ambiguities in intermolecular cross-peak assignments and to gain further understanding into the structural arrangement of these large aggregates.

## Conclusions

The 3D structure of teixobactin in DPC micelles confirms the presence of a C-terminal pyrophosphate binding cage previously suggested in truncated teixobactin analogues. The N-terminal part of the peptide is well-structured in DPC micelles, in contrast to structures of teixobactin analogues in DMSO where it appears mostly disordered. A combination of titrations in solution NMR and solid state NMR experiments reveals that most of the teixobactin is involved in binding lipid II, either *via* interactions or conformational changes upon binding. The pattern of the strongest affected residues is overall very similar in both titrations using water soluble Gram-positive lipid II in the absence of DPC micelles and native Gram-negative lipid II in the presence of DPC micelles, with mostly backbone sites being affected. Residues 2–6 undergo a change from coil to β conformation upon binding of lipid II. We suggest that residues 2–6 are important for the activity due to their involvement in aggregation of teixobactin–lipid II complexes, which in the cell would lead to accumulation of lipid II and inhibition of cell wall synthesis. Importantly, using solid state NMR we have for the first time been able to study the full complex formed between native teixobactin and native lipid II, something that would not be possible with other biophysical techniques such as solution NMR and X-ray crystallography. This study paves the way for more detailed structural analysis of aggregating antibiotics–substrate complexes.

## Experimental

### Sample preparation

Lipid II was synthesized as described previously.^34^,^35^ The biotinylated variant of lipid II used in the FRET experiments was obtained by biotinylation of the *N*-acetylmuramyl-pentapeptide precursor prior to the lipid II synthesis.

Natural abundance teixobactin and [U-^13^C,^15^N]teixobactin were produced the same way by growing *Eleftheria terrae* in R4 growth medium^5^ without (for natural abundance teixobactin) and with (for labeled teixobactin) replacement of labeled materials (purchased from Cambridge Isotope Laboratories, Inc., Tewksbury, MA, USA). The labeled Modified Celtone R4 was prepared as 10 g d-glucose U-^13^C_6_, 1.1 g Celtone base U-^13^C U-^15^N, 0.5 g l-proline U-^13^C_5_^15^N, 10 g MgCl_2_·6H_2_O, 4 g CaCl_2_·2H_2_O, 0.2 g K_2_SO_4_, 5.6 g *N*-[tris(hydroxymethyl)methyl]-2-aminoethanesulfonic acid (TES), and deionized H_2_O to 1 L and pH adjusted to 7.0 using KOH. 1 L of labelled media was inoculated with biomass from an agar plate and grown at 28 °C for 7 days with shaking. The fermentation was centrifuged to separate the cell biomass from the supernatant. The supernatant was decanted into a new container and extracted with an equal volume of butanol, while the cell pellet was extracted with acetone and centrifuged. The acetone and butanol extracts were combined and evaporated to dryness. The resulting residue was dissolved in DMSO and separated by HPLC (water/acetonitrile/0.1% TFA). Of the forty eight fractions generated, fraction 23 contained the majority of the target compound. This fraction was then examined by LC-MS and the expected mass for fully ^13^C and ^15^N labelled teixobactin was the major ion peak ([M + H] = 1315.9). This fraction was dried down, resulting in 10 mg of the desired product.

### Solution NMR

For preparation of TXB_DPC_ [U-^13^C,^15^N]teixobactin was dissolved to a concentration of 2 or 3 mM in 10 mM NaP buffer, pH 6.5, with 100 or 150 mM d_38_ DPC (Eurisotop). For preparation of TXB_aqueous_ [U-^13^C,^15^N]teixobactin was dissolved to a concentration of 0.3 mM in 10 mM NaP buffer, pH 6.5. 2,2-Dimethyl-2-silapentane-5-sulfonate (DSS) was used as internal reference. Lipid II was dissolved in 10 mM NaP buffer, pH 6.5, with 150 mM d_38_ DPC. Water soluble lipid II was dissolved in 10 mM NaP buffer, pH 6.5. All solution NMR experiments were performed on a Bruker Avance II 700 MHz spectrometer equipped with a cryo-probe, using 3 mm NMR tubes. Experiments were performed at 25 °C, additional ^1^H-^15^N and ^1^H-^13^C correlation experiments were performed at 37 °C so that assignments could be compared with assignments obtained in solid state NMR experiments. Initial assignments for TXB_DPC_ were obtained from a natural abundance teixobactin (prepared in the same way as labelled teixobactin) using 2D experiments: ^1^H-^13^C HSQC, ^1^H-^15^N SOFAST HMQC (0.3 s recycle delay), ^1^H-^1^H TOCSY (70 ms mixing time), ^1^H-^1^H COSY. Assignments were confirmed and completed from 3D experiments using [U-^13^C,^15^N]teixobactin: BEST HNCACB (0.3 s recycle delay), CCH-TOCSY (16.3 ms DIPSI-3 mixing time), CBCA(CO)NH (25% non-uniform sampling (NUS) reconstructed in TopSpin using the MDD algorithm^36^). The assignments were referenced to DSS using an external reference. A 3D ^15^N HSQC-NOESY (200 ms mixing time) and a 2D ^1^H-^1^H NOESY (200 ms mixing time) were used for distance restraints in the structure calculations. ^15^N *T*_1_ and *T*_2_ relaxation times were measured using interleaved pseudo 3D experiments. Relaxation delays for ^15^N *T*_1_ experiments (s): 0.005, 0.075, 0.1, 0.2, 0.3, 0.4, 0.5, 0.65, 0.8, 1, 1.25, 1.5. Relaxation delays for ^15^N *T*_2_ experiments (s): 0.017, 0.034, 0.051, 0.068, 0.085, 0.102, 0.136, 0.170, 0.204, 0.254, 0.339. Titrations with native lipid II to [U-^13^C,^15^N]teixobactin were measured using ^1^H-^15^N SOFAST HMQC (0.3 s recycle delay) and ^1^H-^13^C HSQC. Lipid II with a concentration of 15 mM was titrated to the following concentrations (mM): 0.33, 0.79, 1.5, 2, 2.7, 3, 3.4, 3.7, 4, 4.3, 4, leading to a dilution of teixobactin to a final concentration of 2 mM from an initial concentration of 3 mM. Assignments for TXB_aqueous_ were initially transferred from TXB_DPC_ using 2D experiments: ^1^H-^15^N SOFAST HMQC (0.3 s recycle delay) and ^1^H-^13^C HSQC. The assignments were completed using 3D experiments: HNCA and CCH-TOCSY (16.3 ms DIPSI-3 mixing time). The assignments were referenced internally to DSS. ^15^N *T*_1_ and *T*_2_ relaxation times were measured using interleaved pseudo 3D experiments. Relaxation delays for ^15^N *T*_1_ experiments (s): 0.075, 0.1, 0.2, 0.3, 0.4, 0.6, 0.8, 1.1, 1.4. Relaxation delays for ^15^N *T*_2_ experiments (s): 0.017, 0.034, 0.068, 0.102, 0.136, 0.170, 0.237, 0.305. Titrations of water soluble lipid II to [U-^13^C,^15^N]teixobactin were measured using ^1^H-^15^N SOFAST HMQC and ^1^H-^13^C HSQC. Water soluble lipid II with a concentration of 2.4 mM was titrated to the following concentrations (mM): 0.05, 0.1, 0.14, 0.19, 0.23, leading to a dilution of teixobactin from 0.3 to 0.27 mM.

### Solid state NMR

After titrations of lipid II to [U-^13^C,^15^N]teixobactin (in DPC micelles) the sample was transferred from the NMR tube, 10 mM NaP buffer pH 6.5, with 2% DSS was added up to 500 μL leading to a final concentration of approximately: 0.6 mM [U-^13^C,^15^N]teixobactin, 1.4 mM lipid II, 45 mM d_38_ DPC. The sample was sedimented by ultracentrifugation (Beckmann Coulter Optima MAX-XP Ultracentrifuge) for 46 hours at 700 000 × *g*, forming a solid paste. Most of the liquid was removed and a small amount of 10 mM NaP buffer containing 2 mM gadolinium diethylenetriaminepentaacetic acid bismethylamide (Gd(DTPA-BMA)) and 2% DSS was added to the sediment. The sediment was packed into a 0.81 mm Samoson rotor. To keep the sample hydrated during packing small amounts of buffer with Gd(DTPA-BMA) and DSS were added to the rotor. All experiments were recorded at a 600 MHz Bruker Avance II spectrometer using a Samoson HXY 0.81 mm probe at 90 kHz magic angle spinning and a sample temperature of 39 ± 2 °C measured from the water peak referenced to DSS. Proton detection with 30 ms acquisition time was used for all experiments. The addition of Gd(DTPA-BMA) enabled a recycle delay of 0.5 s. Water suppression was achieved by 100–150 ms slpTPPM^37^ at 22.5 kHz nutation frequency (¼ of the spinning speed). The following spectra were acquired: 2D ^1^H-^13^C inverse CP (0.4 ms CP between ^1^H-^13^C and ^13^C-^1^H) 2D ^1^H-^15^N inverse CP (1 ms CP between ^1^H-^15^N and 0.9 ms between ^15^N-^1^H), 3D hCANH (0.4 ms CP between ^1^H-^13^C, 11 ms between ^13^C-^15^N, 0.9 ms between ^15^N-^1^H), 3D hCONH (2.5 ms CP between ^1^H-^13^C, 11 ms between ^13^C-^15^N, 0.9 ms between ^15^N-1H). 3D hCCH TOCSY (0.4 ms CP between ^1^H-^13^C and ^13^C-^1^H, 16.3 ms DIPSI-3 mixing time at 10 kHz nutation frequency) 3D hCOCAHA DREAM (2.5 ms CP between ^1^H-^13^CO, 0.4 ms CP between ^13^CA-^1^HA, 7 ms DREAM between ^13^CO-^13^CA at 45 kHz nutation frequency, ½ of the spinning speed).

### NMR data analysis

TopSpin 3.2 was used to process all spectra. The spectra were assigned in NMRFAM Sparky.^38^ Structure calculation of TXB_DPC_ was performed using UNIO 10 with Cyana 2.1 39 as molecular dynamics software using NOEs as structural restraints. Raw spectra (^1^H-^1^H 2D NOESY and ^15^N 3D HSQC-NOESY) were used as input into the structure calculation. Automatic peak picking and NOE assignments were achieved by UNIO ATNOS-CANDID.^40^,^41^ Cyana library entries for d-amino acids were produced using CyLib:^42^d-glutamine (converted from DGN.cif), d-allo-isoleucine (converted from 28J.cif), d-threonine (converted from DTH.cif). A Cyana library entry for *N*-methylated-d-phenylalanine was initially converted by Cylib from ZAE.cif and slightly modified by producing a .cor file in Cyana containing the new ZAE residue as the first residue and a peptide bond to another amino acid. The amide proton was added in UCSF Chimera^43^ and a pdb file was exported and read in MOLMOL^44^ where coordinates were exported as a library file. The library file was edited manually in a text editor to fit to the Cyana library format. The .cif files were obtained from ; http://www.bpc.uni-frankfurt.de/guentert/wiki/index.php/Cyana_Residue_Library_Entries, except for d-allo-isoleucine (28J.cif), which was obtained from ; http://www.ebi.ac.uk/pdbe-srv/pdbechem/chemicalCompound/show/28J. A Cyana library entry for allo-enduracididine was produced from a drawing of the chemical structure in ChemSketch (ACDLabs Freeware 2012) including peptide bonds to residues before and after. The chemical structure was exported as a .mol file and converted to PDB in UCSF Chimera.^43^ MOLMOL^44^ was used to write coordinate files and the Cyana library entry was finalized by manually rearranging the atoms, adding pseudo atoms and torsion angles in a text editor (PSPAD editor).

Peak integrals from TopSpin were exported to MatLab where a single decaying exponential function was used to fit the *R*_1_ and *R*_2_ relaxation data. Errors were calculated by a Monte Carlo error estimation. A random number between 0 and 1 was multiplied with the integral error, which was based on the noise level compared to the signal for each peak, and added to the recalculated integrals. The fitting was then repeated 2000 times with a new random number between 0 and 1 generated each time. Two times the standard deviations of the *R*_1_ or *R*_2_ values received from the fits for each residue were used as errors.

The correlation times (*τ*_c_) for TXB_aqueous_ and TXB_DPC_ were calculated from the ^15^N *T*_1_ and *T*_2_ relaxation times and the ^15^N resonance frequency (*ν*_N_), 71 MHz at a 16.4 T NMR magnet (700 MHz ^1^H Larmor frequency) using eqn (1):1
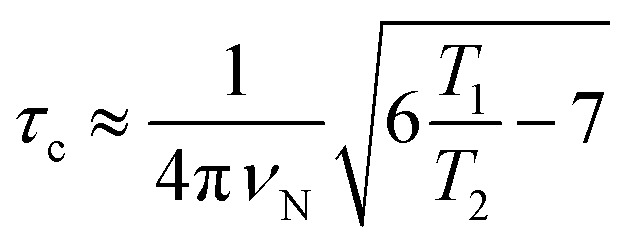



which is generally used for rigid protein molecules in the limit of slow molecular motion (*τ*_c_ ≫ 0.5 ns), using high magnetic field (^1^H Larmor frequency ≥ 500 MHz).^45^

To determine binding affinities the peak intensities of unbound teixobactin (TXB_DPC_ or TXB_aqueous_) were measured in the titration spectra and fitted to eqn (2) (based on eqn (6) in ref. 25):2
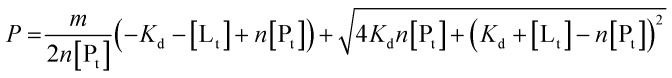
where *P* is the normalized peak intensity, *m* = 1 if the point without an added ligand is included and *m* is fitted if the point without an added ligand is not included, *n* is the molar ratio for binding (lipid II/teixobactin), [L_t_] is ligand concentration (lipid II), [P_t_] is protein concentration (teixobactin). The data points used for the fitting were: 0.33, 0.79, 1.5, 2, 2.7, 3 (mM) lipid II for TXB_DPC_ and 0.05, 0.1, 0.14, 0.19, 0.23 (mM) water soluble lipid II for TXB_aqueous_. The fitting of the data was done by minimization of the *χ*^2^ target function (eqn (3)):3
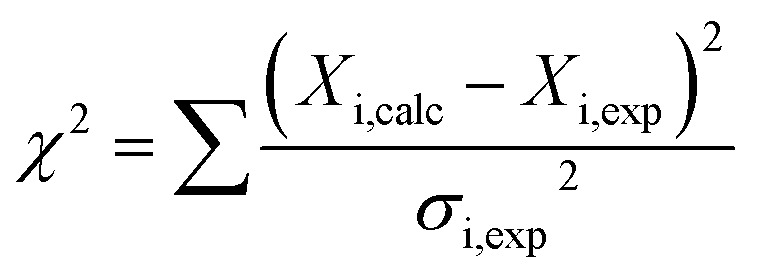
where *X*_i_ are the data sets and *σ*_i_ the corresponding error. Errors for the *K*_d_ fits were calculated in the same way as for the relaxation data using 500 steps in the Monte Carlo error estimation.

Chemical shift perturbations were calculated as Euclidian distances^25^ (eqn (4)):4
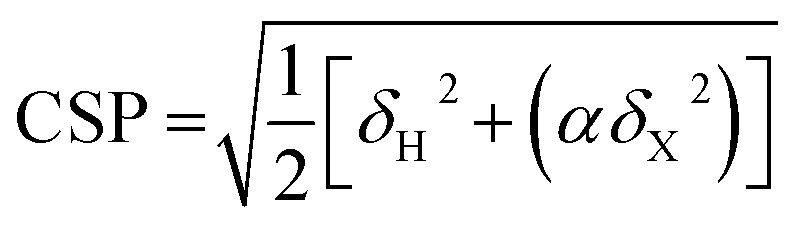
where *δ* is the difference in chemical shift between TXB_aqueous_ and TXB_DPC_ or between TXB_DPC_ and TXB_lipid II_, *α* = 0.14 if *x* = ^15^N and *α* = 0.3 if *x* = ^13^C.

FRET. 1 μM biotinylated lipid II was labelled with streptavidin-conjugated FRET dyes (HTRF, CisBio) and incubated with ramoplanin, teixobactin, deoxyactagardine B, mersacidin and vancomycin at concentrations ranging between 0.5 and 32 μM. Samples were incubated for 30 min at ambient temperature and FRET magnitude was measured in a time-resolved manner, using the Clariostar plate reader (BMG Labtech). The experiments were performed in triplicates. Full reaction composition: 1 μM biotin–DAP–lipid II, 1 × HTRF labelling mixture (terbium cryptate, d2), 50 mM bis–Tris propane pH 8.5, 10 mM MgCl_2_, 20 mM NaCl, 0.05% Triton X-100, 1% glycerol.

## Author contributions

C. Ö. and J. R. L. designed and analysed the NMR experiments and wrote the paper, C. Ö. performed the NMR experiments, C. Ö. and T. H. performed the NMR structure calculation. G. P. W. and C. G. D. designed and analysed the FRET experiments, G. P. W. performed the FRET experiments. C. G. D. and A. J. L. optimized lipid II synthesis and analysis, C. Ö., G. P. W., A. C. C., J. A. T. and A. J. L. produced and characterised lipid II. D. E. H., A. L. S. and A. J. P. produced and characterised the natural abundance teixobactin and [U-^13^C,^15^N]teixobactin. The manuscript was written through contributions from all authors.

## Conflicts of interest

The following authors, A. J. Peoples, A. L. Spoering, D. E. Hughes, and K. Lewis declare competing financial interests as they are employees and consultants of NovoBiotic Pharmaceuticals.

## Data availability

NMR assignments were deposited in the BMRB under accession numbers 27478 (TXB_DPC_), 27479 (TXB_aqueous_) and 27480 (TXB_lipid II_). Raw NMR data are available from ; http://wrap.warwick.ac.uk/108554/.

## Supplementary Material

Supplementary information

Supplementary information
