# Supplementary material for: Structural studies suggest aggregation as one of the modes of action for teixobactin
Source: Chem Sci. 2018 Sep 20;9(47):8850–9. doi: 10.1039/c8sc03655a (PMC6296168; doi:10.1039/c8sc03655a)
Supplement: Supplementary file 1 [file SC-009-C8SC03655A-s001.pdf]

Electronic Supplementary Material (ESI) for Chemical Science.
This journal is © The Royal Society of Chemistry 2018

Electronic Supplementary Information for

"Structural studies suggest aggregation as one of the modes of action for teixobactin"

Carl Öster[a], Grzegorz P. Walkowiak[a, b], Dallas E. Hughes[c], Amy L. Spoering[c], Aaron J. Peoples[c], Anita C. Catherwood[b], Julie A. Tod[b], Adrian J. Lloyd[b], Torsten Herrmann[d], Kim Lewis[e], Christopher G. Dowson[b], Józef R. Lewandowski[a*]

[a]Department of Chemistry, University of Warwick, Coventry, CV4 7AL, U.K.
[b]School of Life Sciences, University of Warwick, Coventry, CV4 7AL, U.K.
[c]NovoBiotic Pharmaceuticals, Cambridge, MA02138, USA
[d]Centre de Résonance Magnétique Nucléaire à Très Hauts Champs, Institut des Sciences Analytiques (UMR 5280 – CNRS, Ecole Normale Supérieure de Lyon, Université Claude Bernard Lyon 1), Université de Lyon, 69100 Villeurbanne, France
[e]Antimicrobial Discovery Center, Northeastern University, Department of Biology, Boston, Massachusetts 02115, USA

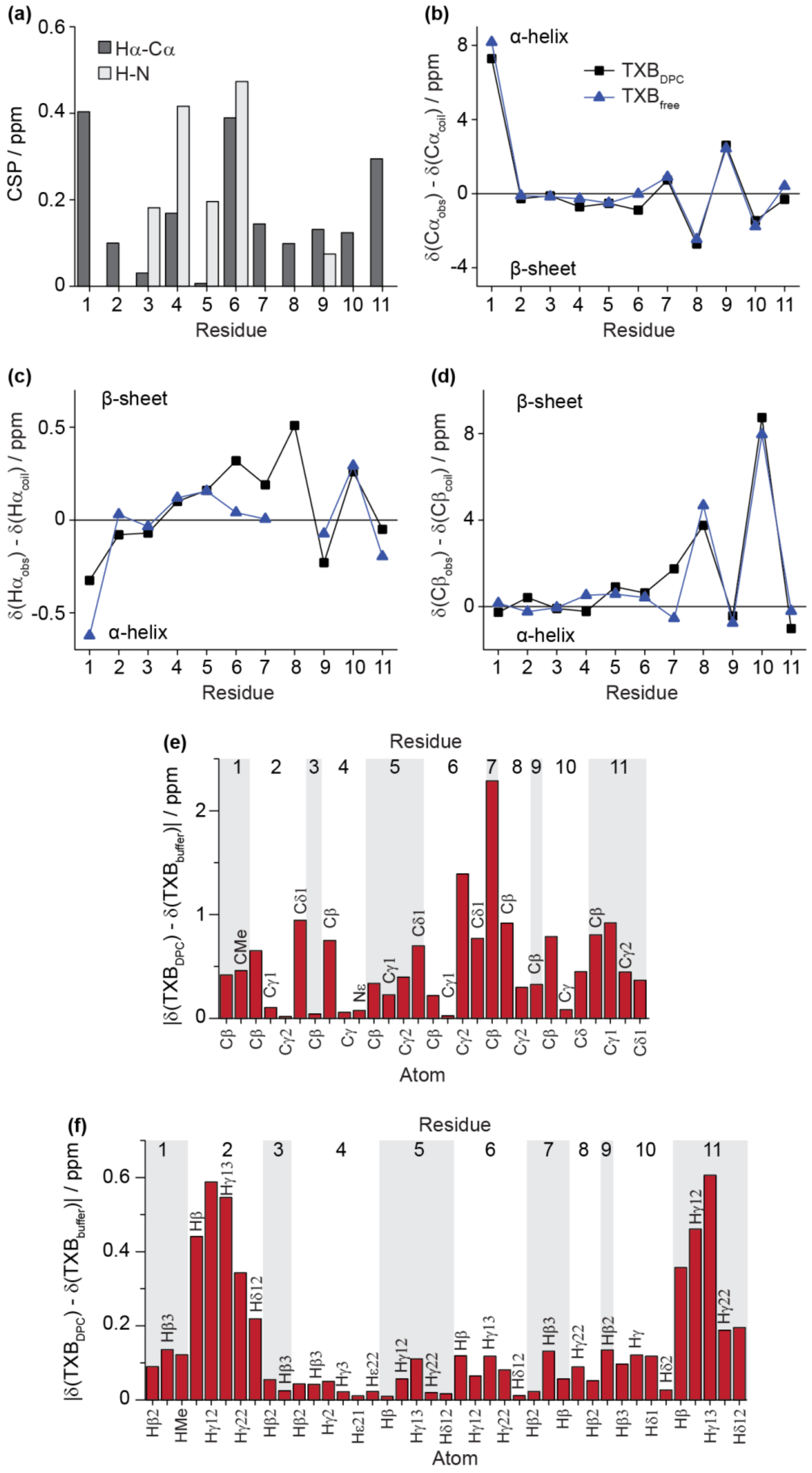

Figure ESI1. Comparison of chemical shifts between teixobactin in phosphate buffer in the presence and absence of DPC micelles. (a) Chemical shift perturbations (CSP). (b) C^{α} secondary chemical shifts. (c) H^{α} secondary chemical shifts. (d) C^{β} secondary chemical shifts. (e) Chemical shift differences for side-chain carbons and nitrogens. (f) Chemical shift differences for side-chain protons.

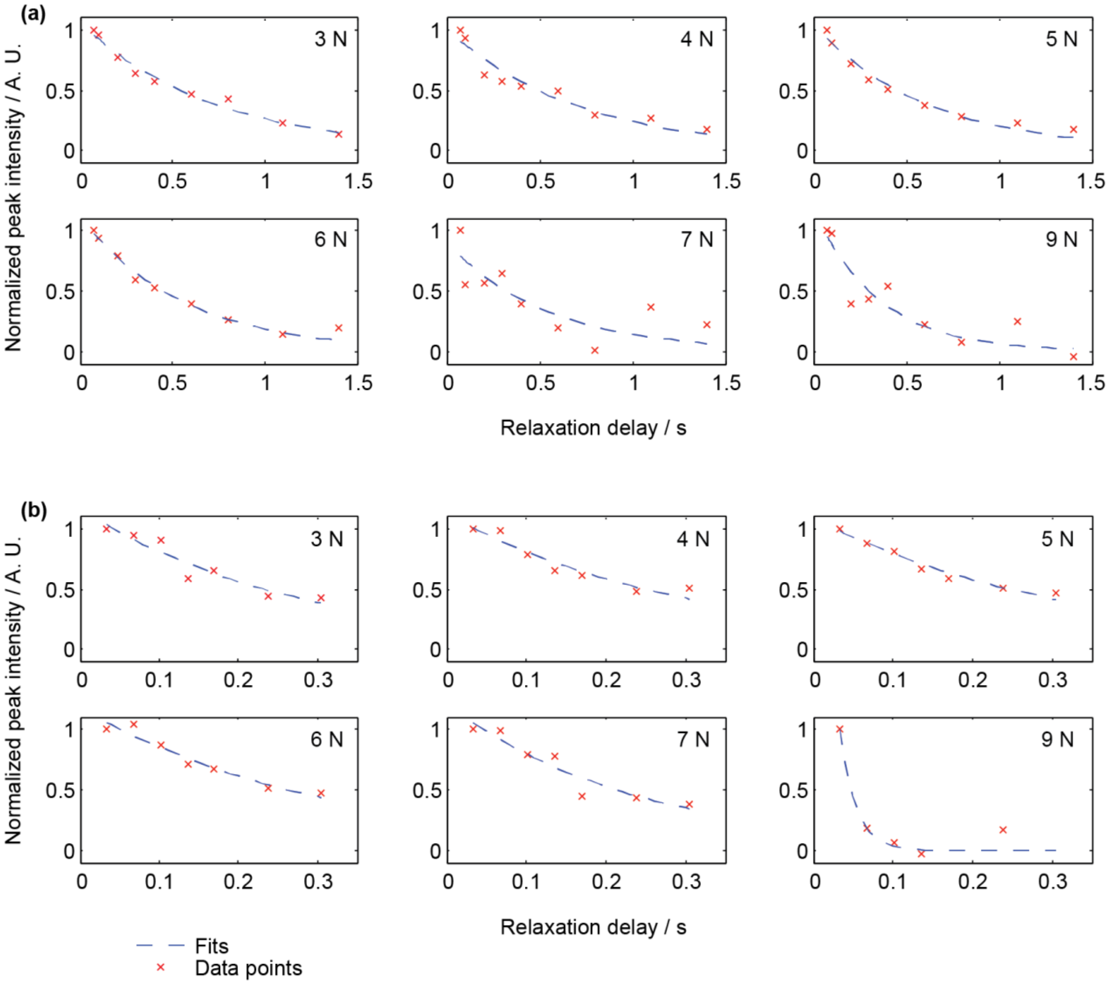

Figure ESI2. Solution NMR ^{15}N R_1 (a) and R_2 (b) relaxation measurements for teixobactin in aqueous solution (red crosses). Dashed blue lines indicate the best fits to a single decaying exponential. Data obtained at 700 MHz ^{1}H Larmor frequency and a sample temperature of 25 ˚C.

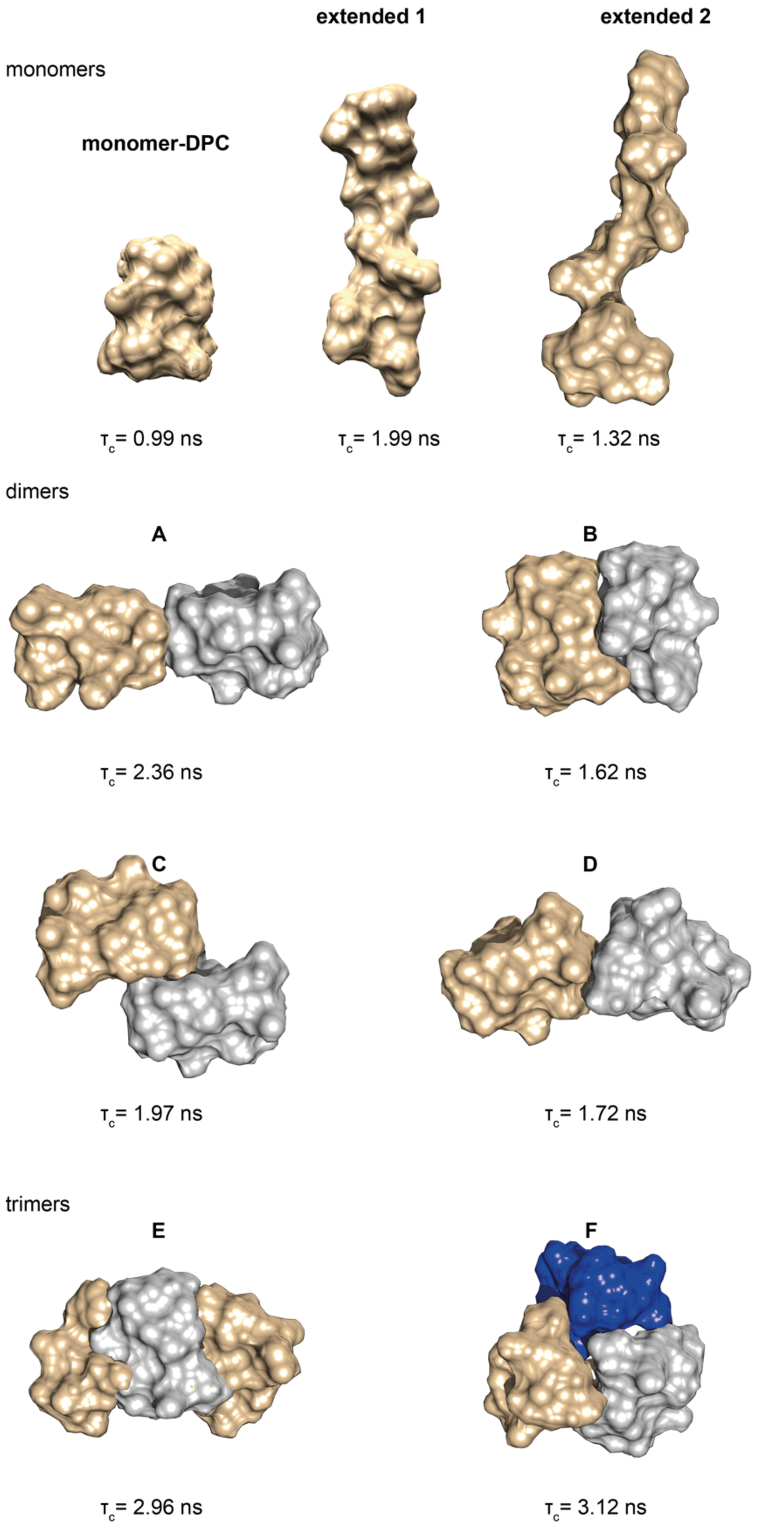

Figure ESI3. Models of potential dimers and trimers based on the NMR structure of teixobactin. Correlation times were predicted using HYDRONMR[1] based on each of the model and are compared with correlation times calculated from ^{15}N T_1 and T_2 relaxation times in SI table 3.

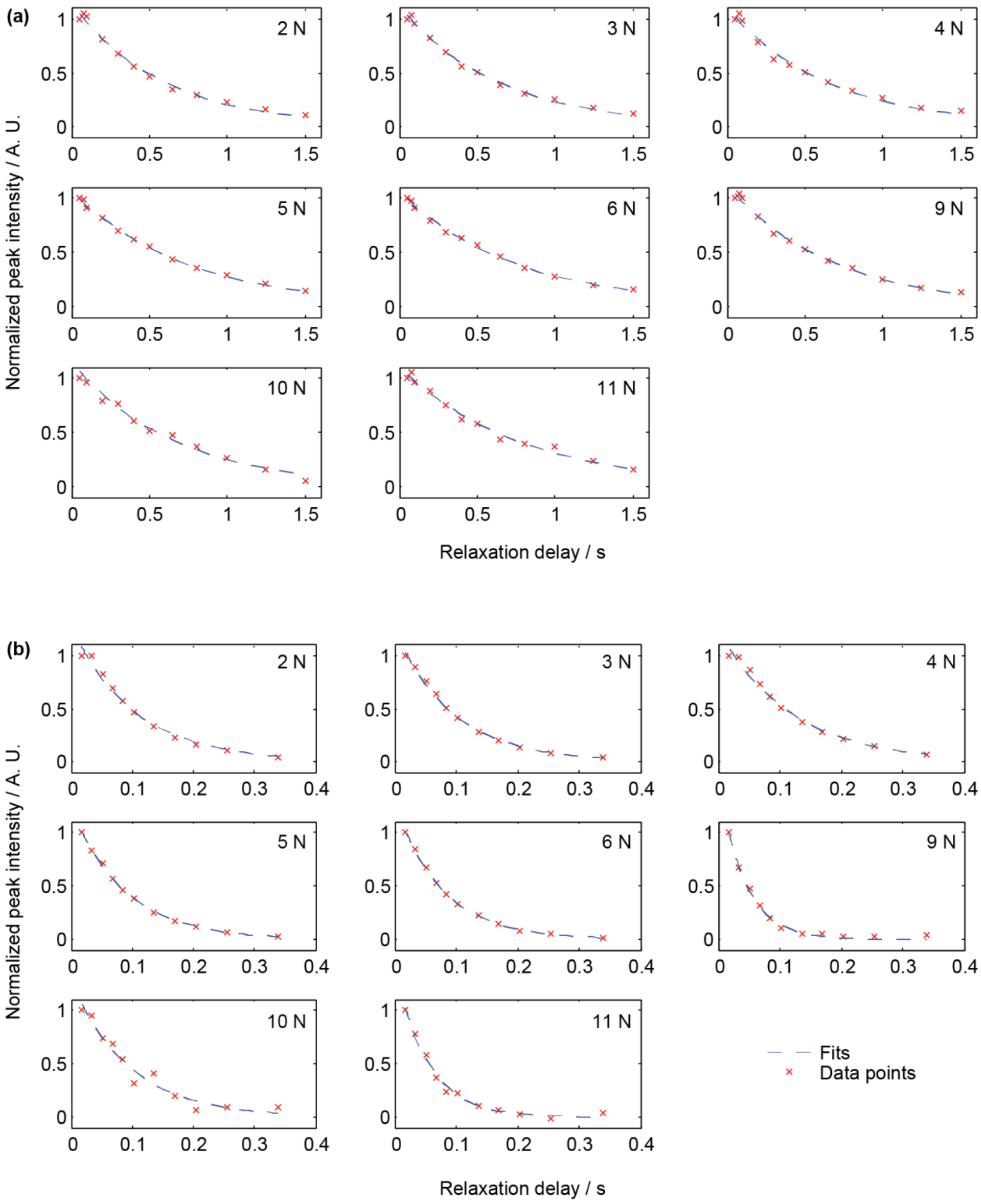

Figure ESI4. Solution NMR ^{15}N R_1 (a) and R_2 (b) relaxation measurements for teixobactin in DPC micelles (red crosses). Dashed blue lines indicate the best fits to a single decaying exponential. Data obtained at 700 MHz ^{1}H Larmor frequency and a sample temperature of 25 ˚C.

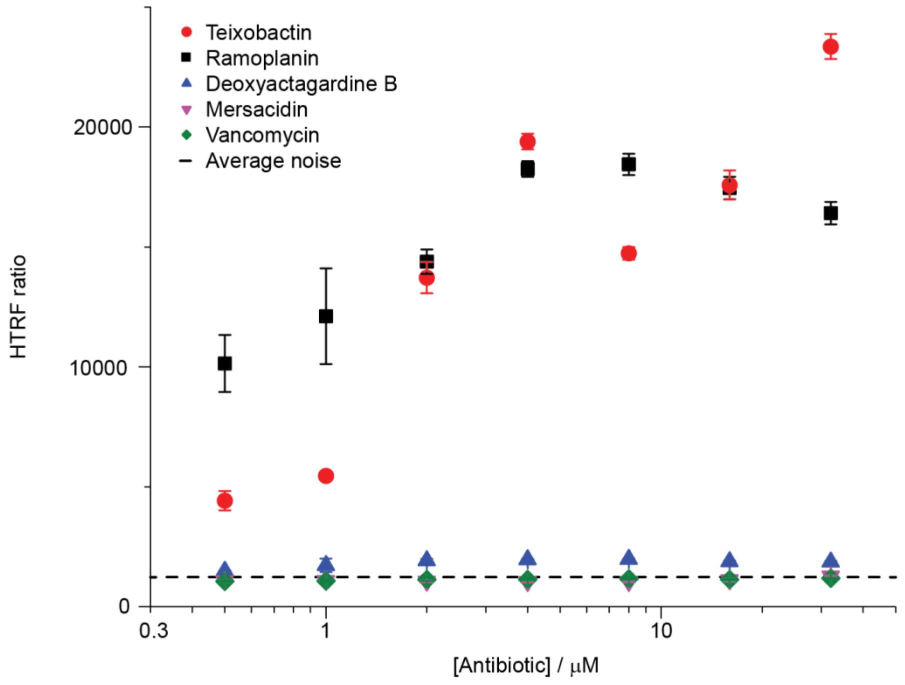

Figure ESI5. Evolution of FRET signal in presence of lipid II-binding antibiotics. Data points represent mean values of 3 replicates. Error bars show standard deviation. The high HTRF (Homogeneous Time-Resolved Fluorescence) ratio is indicative of aggregation.

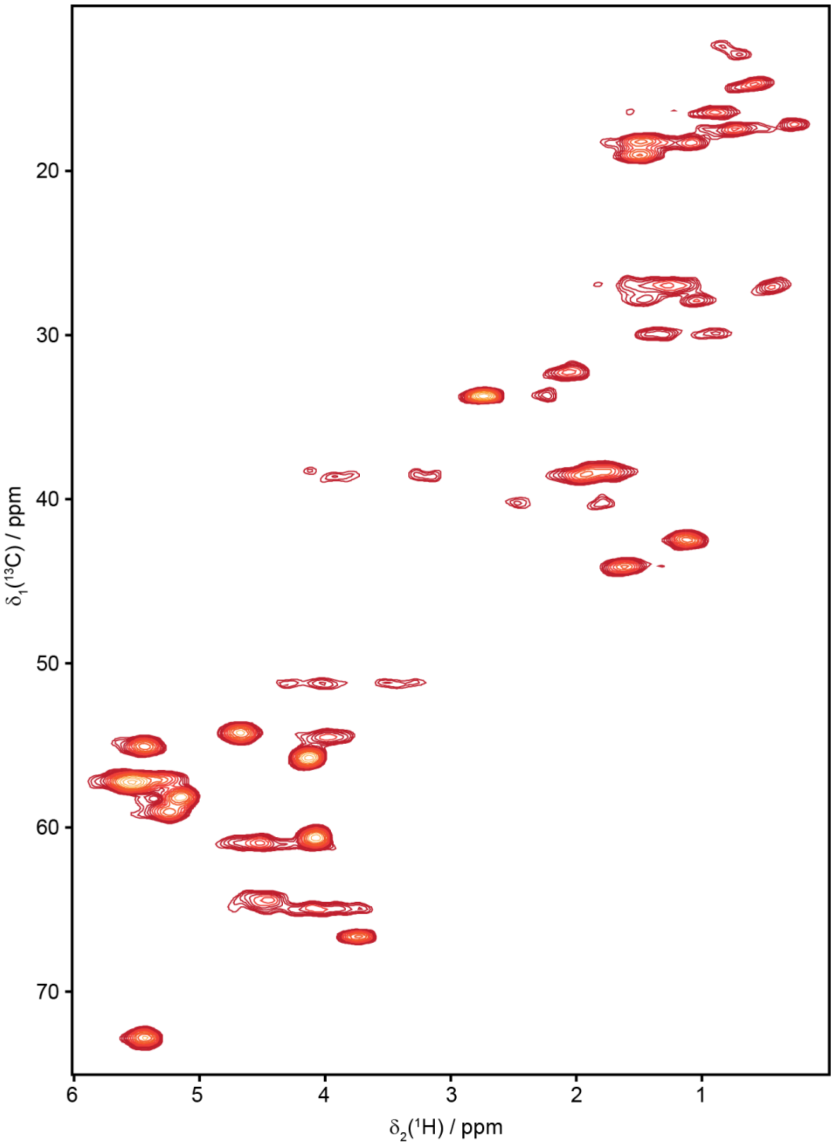

Figure ESI6. ^{1}H detected cross-polarisation based 2D ^{1}H-^{13}C solid state NMR correlation spectrum of sedimented [U^{13}C-^{15}N]teixobactin in complex with natural abundance Gram-negative lipid II in DPC micelles. Data acquired at 600 MHz ^{1}H Larmor frequency and 90 kHz magic angle spinning frequency.

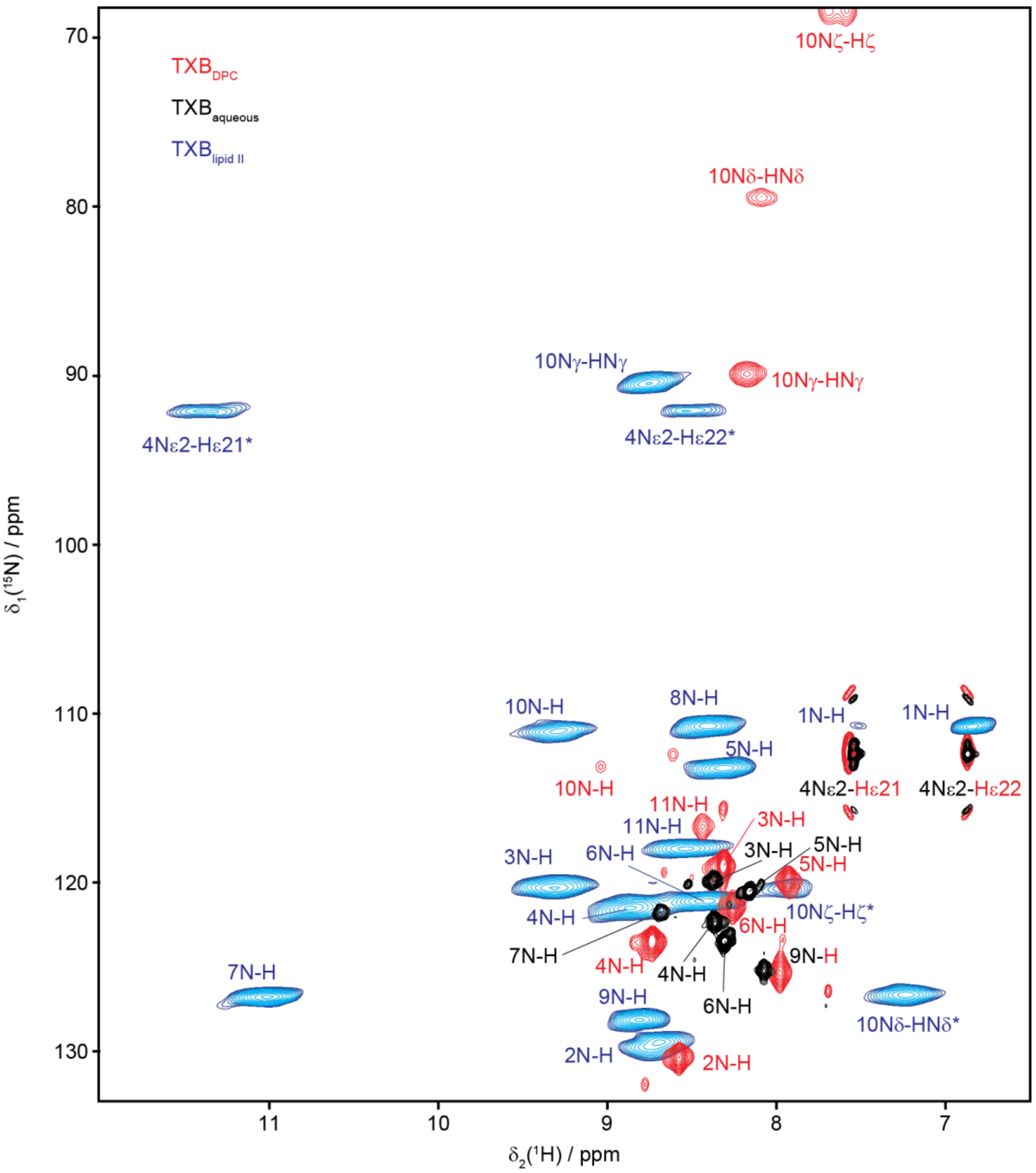

Figure ESI7. Overlay of 2D ^{1}H-^{15}N correlation spectra of [U^{13}C-^{15}N]teixobactin in aqueous solution in DPC micelles (TXB$_{DPC}$; red), [U^{13}C-^{15}N]teixobactin in aqueous solution in the absence of DPC micelles (TXB$_{aqueous}$; black) and solid-state sedimented complex of U^{13}C-^{15}N]teixobactin with lipid II in the presence of DPC micelles (TXB$_{lipid\ II}$; blue). Solution NMR data were acquired at a 700 MHz spectrometer and solid-state NMR data were obtained at 600 MHz ^{1}H Larmor frequency and 90 kHz magic angle spinning frequency. The sedimented sample was prepared from a solution NMR sample used for titrations of teixobactin with lipid II.

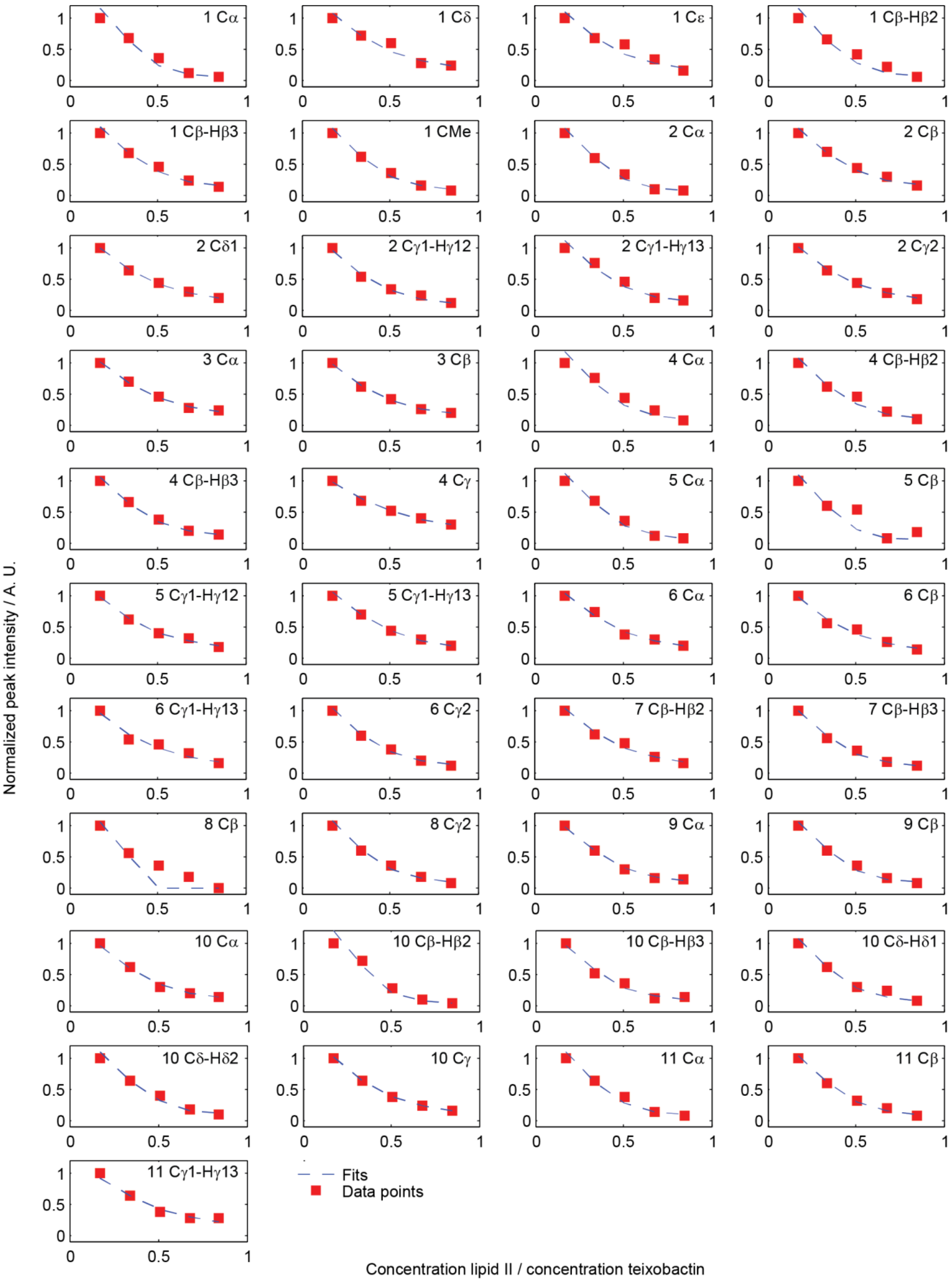

Figure ESI8. NMR titration of teixobactin with water soluble Gram-positive lipid II performed in aqueous solution. Red squares represent experimental data points. Dashed blue lines represent the best fits to equation 2 (see Methods section) assuming 2:1 binding of teixobactin to lipid II. The best fit values for local apparent K_d are given in SI table 4. The data were acquired using ^{1}H-^{13}C HSQC spectra at 700 MHz ^{1}H Larmor frequency and a sample temperature of 25 ˚C.

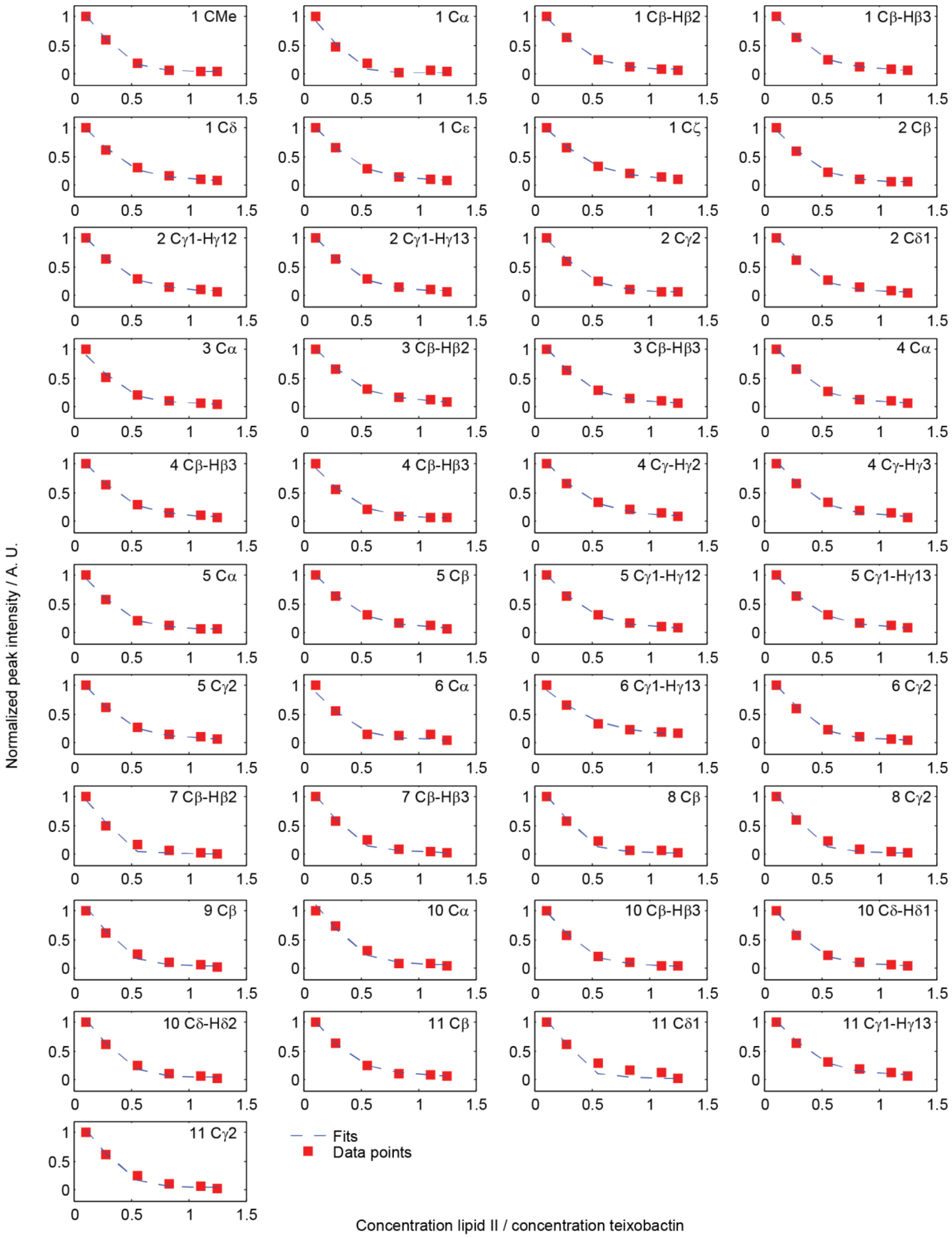

Figure ESI9. NMR titration of teixobactin with Gram-negative lipid II in DPC micelles. Red squares represent experimental data points. Dashed blue lines represent the best fits to equation 2 (see Methods section) assuming 2:1 binding of teixobactin to lipid II. The best fit values for local apparent K_d are given in SI table 5. The data were acquired using ^{1}H-^{13}C HSQC spectra at 700 MHz ^{1}H Larmor frequency and a sample temperature of 25 ˚C .

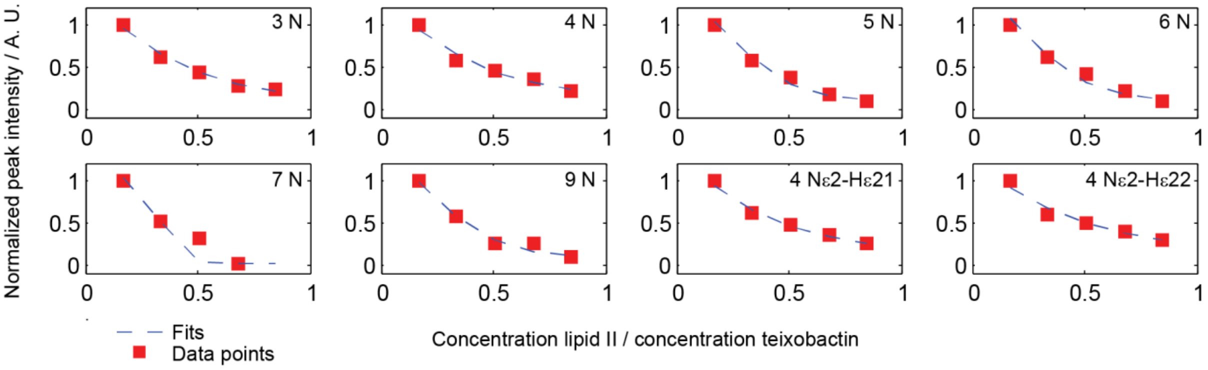

Figure ESI10. NMR titration of teixobactin with water soluble Gram-positive lipid II in aqueous solution. Red squares represent data points. Dashed blue lines represent the best fits to equation 2 (see Methods section) assuming 2:1 binding of teixobactin to lipid II. The best fit values for local apparent K_d are given in SI table 4. The data were acquired using ^{1}H-^{15}N SOFAST-HMQC spectra at 700 MHz ^{1}H Larmor frequency and a sample temperature of 25 ˚C.

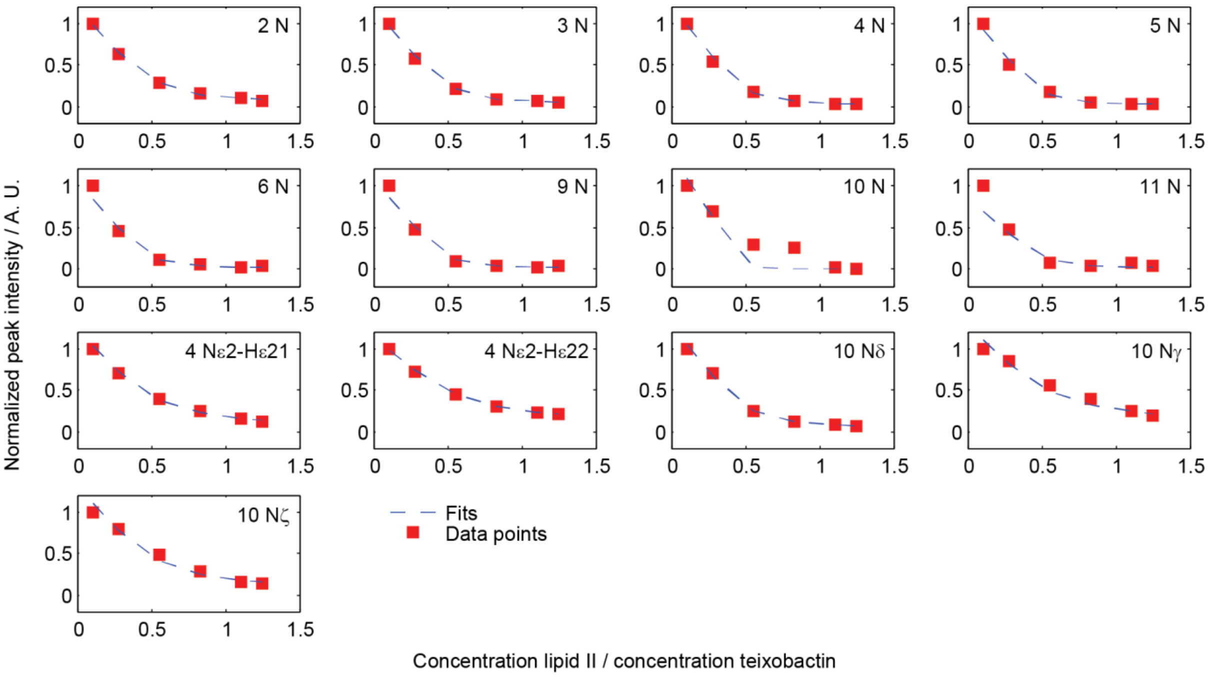

Figure ESI11. NMR titration of teixobactin with Gram-negative lipid II in DPC micelles. Red squares represent data points. Dashed blue lines represent the best fits to equation 2 (see Methods section) assuming 2:1 binding of teixobactin to lipid II. The best fit values for local apparent K_d are given in SI table 5. The data were acquired using ^{1}H-^{15}N SOFAST-HMQC spectra at 700 MHz ^{1}H Larmor frequency and a sample temperature of 25 ˚C.

Tables

Table ESI1. Details from structure calculation for teixobactin in DPC micelles, performed using UNIO ATNOS-CANDID[2,3] with CYANA[4] as molecular dynamics software.

Assigned cross-peaks		$^{1}H – ^{15}N$ 3D HSQC NOESY	$^{1}H – ^{1}H$ 2D NOESY
Total		126	377
Diagonal		0	0
Intraresidual (i = j)		52	197
Sequential (|i - j| = 1)		48	123
Medium range (1 < |i - j| < 5)		24	49
Long range (|i - j| ≥ 5)		2	8
Distance restraints[a]			
Total	235		
Intraresidual (i = j)	69		
Sequential (|i - j| = 1)	96		
Medium range (1 < |i - j| < 5)	58		
Long range (|i - j| ≥ 5)	12		
No. of restraints per residue	21		
Backbone RMSD	< 0.01 Å		
Heavy-atom RMSD	0.26 ± 0.07 Å		

[a] only meaningful, non-redundant distance restraints are reported

Table ESI2. ^{15}N backbone relaxation rates for teixobactin measured at 700 MHz ^{1}H Larmor frequency and a sample temperature of 25 ˚C.

Sample	$TXB_{aqueous}$				TXB_{DPC}			
Residue	R_1 (s^{-1})	error	R_2 (s^{-1})	error	R_2 (s^{-1})	error	R_2 (s^{-1})	error
1								
2					1.720	0.007	9.597	0.043
3	1.411	0.100	3.674	0.209	1.580	0.004	10.552	0.032
4	1.448	0.097	3.289	0.188	1.541	0.004	8.454	0.027
5	1.661	0.040	3.202	0.069	1.352	0.005	11.394	0.045
6	1.778	0.049	3.280	0.088	1.319	0.005	12.935	0.073
7	1.854	0.460	4.142	0.731				
8								
9	2.866	0.655	50.312	25.125	1.506	0.018	23.437	0.620
10					1.526	0.114	10.393	0.958
11					1.275	0.03	18.934	0.708

w

Table ESI3. Correlation times (τ_c) estimated from the relaxation rates in SI table 2, using equation 1 in the Methods section or predicted from the NMR structure of teixobactin as monomer, dimer or trimer using HYDRONMR[1] (see SI figure 4).

Sample	$TXB_{aqueous}$		TXB_{DPC}		Predicted	
Residue	τ_c (ns)	error	τ_c (ns)	error	Model	τ_c (ns)
1					monomer-DPC	0.99
2			5.77	0.03	monomer-extended	1.32-1.99
3	3.29	0.30	6.45	0.03	dimer A	2.36
4	2.89	0.25	5.71	0.02	dimer B	1.62
5	2.40	0.08	7.40	0.04	dimer C	1.97
6	2.26	0.09	8.07	0.06	dimer D	1.72
7	2.84	0.87			trimer E	2.96
8					trimer F	3.12
9	11.12	6.11	10.42	0.30		
10			6.53	0.77		
11			10.16	0.45		

Table ESI4. Local apparent K_d values obtained by fitting the data from titration of teixobactin with water soluble Gram-positive lipid II in aqueous solution, to equation 2 (Methods section). 2:1 binding of teixobactin to lipid II is assumed.

Peak	K_d (μM)	error	Res	K_d (μM)	error
$1C^{Me}$	6.08	0.38	6N	7.35	1.27
$1C^{\alpha}$	3.22	1.81	$6C^{\beta}$	2.78	5.35
$1C^{\beta}$	10.71	3.12	$6C^{\gamma 1}$	24.47	6.91
$1C^{\beta}$	4.36	3.18	7N	0.15	0.90
$1C^{\delta a}$	19.77	10.51	$7C^{\alpha}$	17.15	4.40
$1C^{\varepsilon a}$	15.15	6.54	$7C^{\beta}$	14.33	3.36
$2C^{\beta}$	6.51	3.02	$7C^{\beta}$	8.13	1.92
$2C^{\gamma 1}$	16.72	5.33	$8C^{\beta}$	0.15	0.13
$2C^{\gamma 2}$	9.38	0.77	$8C^{\gamma}$	6.04	0.61
3N	22.40	4.24	9N	7.98	3.09
$3C^{\alpha}$	19.36	3.94	$9C^{\alpha}$	9.22	3.41
$3C^{\beta}$	17.29	1.21	$9C^{\beta}$	5.79	0.48
4N	25.58	4.54	$10C^{\alpha}$	10.54	4.74
$4C^{\alpha}$	5.73	2.16	$10C^{\beta}$	7.33	3.68
$4C^{\beta}$	9.26	3.49	$10C^{\beta}$	1.94	2.37
$4C^{\beta}$	8.26	2.89	$10C^{\gamma}$	12.34	3.40
$4C^{\gamma}$	34.94	6.02	$10C^{\delta}$	4.92	1.78
$4N^{\varepsilon 2}$	39.86	4.73	$10C^{\delta}$	6.86	1.85
$4N^{\varepsilon 2}$	29.74	3.16	$11C^{\alpha}$	5.49	1.70
5N	7.24	1.09	$11C^{\beta}$	12.75	5.41
$5C^{\alpha}$	4.73	2.60	$11C^{\gamma 1}$	9.52	3.74
$5C^{\beta}$	13.73	3.74	$11C^{\gamma 1}$	10.79	5.18
$5C^{\gamma 1}$	17.70	5.01	$11C^{\gamma 2}$	16.33	0.61
$5C^{\gamma 1}$	17.27	4.89	$11C^{\delta 1}$	18.09	0.91

[a] assignments for the aromatics are ambiguous

Table ESI5. Local apparent K_d values obtained by fitting the data from titration of teixobactin with Gram-negative lipid II in DPC micelles to equation 2 (Methods section). 2:1 binding of teixobactin to lipid II is assumed.

Peak	K_d (μM)	error	Peak	K_d (μM)	error
$1C^{Me}$	40.40	0.91	$5C^{\beta}$	125.47	13.44
$1C^{\alpha}$	19.51	16.16	$5C^{\gamma1}$	129.87	8.75
$1C^{\beta}$	109.17	17.40	$5C^{\gamma1}$	136.43	12.76
$1C^{\beta}$	93.81	12.50	$5C^{\gamma2}$	101.21	1.79
$1C^{\delta}$	125.63	35.51	6N	37.43	17.83
$1C^{\varepsilon}$	129.70	19.68	$6C^{\alpha}$	74.13	23.57
$1C^{\zeta}$	189.04	52.89	$6C^{\gamma1}$	287.36	18.66
2N	132.80	14.31	$6C^{\gamma2}$	64.31	1.86
$2C^{\beta}$	84.32	14.86	$7C^{\beta}$	6.13	7.11
$2C^{\gamma1}$	114.68	17.62	$7C^{\beta}$	35.97	23.77
$2C^{\gamma1}$	110.62	11.82	$8C^{\beta}$	25.69	25.46
$2C^{\gamma2}$	78.74	1.24	$8C^{\gamma2}$	26.38	2.78
$2C^{\delta1}$	81.30	1.66	9 N	35.89	19.97
3N	75.76	9.07	$9C^{\beta}$	46.13	2.38
$3C^{\alpha}$	70.77	17.93	10N	2.65	62.07
$3C^{\beta}$	136.05	6.72	$10C^{\alpha}$	64.02	15.16
$3C^{\beta}$	103.31	6.40	$10C^{\beta}$	60.64	50.01
4N	42.68	7.21	$10C^{\delta}$	69.20	15.56
$4C^{\alpha}$	98.62	16.73	$10C^{\delta}$	47.13	10.10
$4C^{\beta}$	116.41	13.34	$10N^{\gamma}$	377.36	86.42
$4C^{\beta}$	90.50	14.99	$10N^{\delta}$	96.62	23.15
$4C^{\gamma}$	147.93	7.80	$10N^{\zeta}$	239.23	50.74
$4C^{\gamma}$	127.06	7.58	11N	52.47	62.05
$4N^{\varepsilon2}$	230.41	11.68	$11C^{\beta}$	93.28	50.38
$4N^{\varepsilon2}$	423.73	21.15	$11C^{\gamma1}$	123.00	11.80
5N	44.39	16.15	$11C^{\gamma2}$	43.69	1.31
$5C^{\alpha}$	85.25	18.97	$11C^{\delta1}$	19.92	1.79

References

1. García de la Torre, J., Huertas, M. . & Carrasco, B. HYDRONMR: Prediction of NMR Relaxation of Globular Proteins from Atomic-Level Structures and Hydrodynamic Calculations. *J. Magn. Reson.* **147,** 138–146 (2000).

2. Herrmann, T., Güntert, P. & Wüthrich, K. Protein NMR structure determination with automated NOE-identification in the NOESY spectra using the new software ATNOS. *J. Biomol. NMR* **24,** 171–89 (2002).

3. Herrmann, T., Güntert, P. & Wüthrich, K. Protein NMR structure determination with automated NOE assignment using the new software CANDID and the torsion angle dynamics algorithm DYANA. *J. Mol. Biol.* **319,** 209–27 (2002).

4. Güntert, P., Mumenthaler, C. & Wüthrich, K. Torsion angle dynamics for NMR structure calculation with the new program Dyana. *J. Mol. Biol.* **273,** 283–298 (1997).
